# Pure Infarct of the Oculomotor Nucleus With Fetal Posterior Cerebral Artery Involvement

**DOI:** 10.7759/cureus.20282

**Published:** 2021-12-08

**Authors:** Myra Ahmad, Salman Ahmad, Hamzah Ahmad, Eric Basile, Patricia Roche

**Affiliations:** 1 Radiology, Touro College of Osteopathic Medicine, New York, USA; 2 Radiology, Stony Brook University, Stony Brook, USA

**Keywords:** posterior circulation stroke, oculomotor apraxia, neuro radiology, circle of willis variants, pca infarction

## Abstract

Vascular anomalies are present in the posterior circulation. In the case of this stroke patient, the posterior cerebral artery (PCA) was shown to have a fetal origin. A fetal PCA is classified as either a partial or complete fetal PCA, which can be determined by the presence of a remnant or absence of P1, the PCA segment directly arising from the terminal of the basilar artery. If absent, the PCA has arisen completely from the internal carotid artery (ICA) and is termed complete fetal PCA, or cfPCA. A partial fetal PCA, or pfPCA, is what is found when a hypoplastic segment persists. Here, we report a partial infarction of the oculomotor nucleus with ipsilateral fetal PCA in a 59-year-old female.

## Introduction

The CDC reports cerebrovascular accidents (CVAs), or strokes, as the fifth leading cause of death in all Americans annually [1]. Every 40 seconds, an American will have a stroke, and every 4 minutes stroke-associated mortality occurs [2]. An estimated 147,000 people die from CVAs every year. Overall, 7.8 million living adults in the United States have been affected by a stroke, amounting to 3.1% of the total population, resulting in nearly two million office visits a year and half a million visits to the ER [2]. Strokes are a leading cause of long-term disability, and nearly half of all strokes in patients 65 years and older will cause reductions in mobility. Common risk factors for stroke include smoking, obesity, hypertension, and hyperlipidemia, with one in three adults having at least one or a combination of these factors [2]. The majority of strokes are ischemic in etiology, i.e., some form of vascular occlusion is preventing blood flow to a portion of the brain or brainstem, leading to rapid tissue death should the vascular insult not be immediately addressed.

Posterior circulation strokes represent 20% of all strokes [3]. It is defined as the blood flow from the vertebral and basilar arteries, up to and including the vascular territory of the posterior cerebral artery (PCA). Common symptoms of strokes involving the PCA include ocular apraxia, ptosis, pupillary dilation, and the classic "down and out" appearance of the ipsilateral eye, along with contralateral muscle weakness, hyperreflexia, and ipsilateral facial numbness [4]. Interestingly, Mateos et al. note that contralateral superior extraocular motion would be restricted or absent as well, as the superior rectus subnucleus within the oculomotor nucleus is supplied contralaterally [5].

Vascular anomalies are present in the posterior circulation. Here, we report a fetal origin of the PCA seen in a stroke patient. A fetal PCA is classified as either a partial or complete fetal PCA. This is determined by whether P1, the PCA segment directly arising from the terminal of the basilar artery, is hypoplastic or absent. If absent, the PCA has arisen completely from the ICA and is termed complete fetal PCA, or cfPCA. If a hypoplastic segment remains, however, it is known as a partial fetal PCA, or pfPCA [6]. Shaban et al. showed in a retrospective analysis from 2008 to 2010 that up to 9.5% of patients had cfPCA, while pfPCA was prevalent in 15.1% of patients [6]. We report here a partial infarction of the oculomotor nucleus with ipsilateral fetal PCA in a 59-year-old female.

## Case presentation

A 59-year-old female with a past medical history of uncontrolled hypertension presented to the emergency department with sudden onset of double vision starting that evening. Her only surgical history was one uncomplicated C-section years prior. The patient denied tobacco or illicit drug use but admitted to consuming three to four alcoholic beverages daily. She denied having similar symptoms previously and stated that the double vision had not worsened or resolved since its onset. She stated that closing one eye alleviated the vision changes. The patient presented to the emergency department from outpatient ophthalmology due to suspicion of a cerebrovascular event. 

At the time of presentation, she denied dizziness, headache, impaired speech, dysphagia, difficulty ambulating, weakness, or sensory changes. The patient had not experienced recent fevers, coughs, chills, chest pain, shortness of breath, nausea, vomiting, diarrhea, or constipation. Pertinent physical findings include limited medial gaze of the right eye, exotropia on frontward gaze, anisocoria with the left pupil being larger in diameter than the right, mild ocular reactivity to light, and pupillary dilation believed to be due to a dilated eye examination conducted by the ophthalmologist. The remaining extraocular muscles were intact and there was no nystagmus appreciated. Cranial nerves two through 12 were grossly intact aside from the medial rectus palsy as noted above. The rest of the physical examination was unremarkable. 

**Figure 1:**
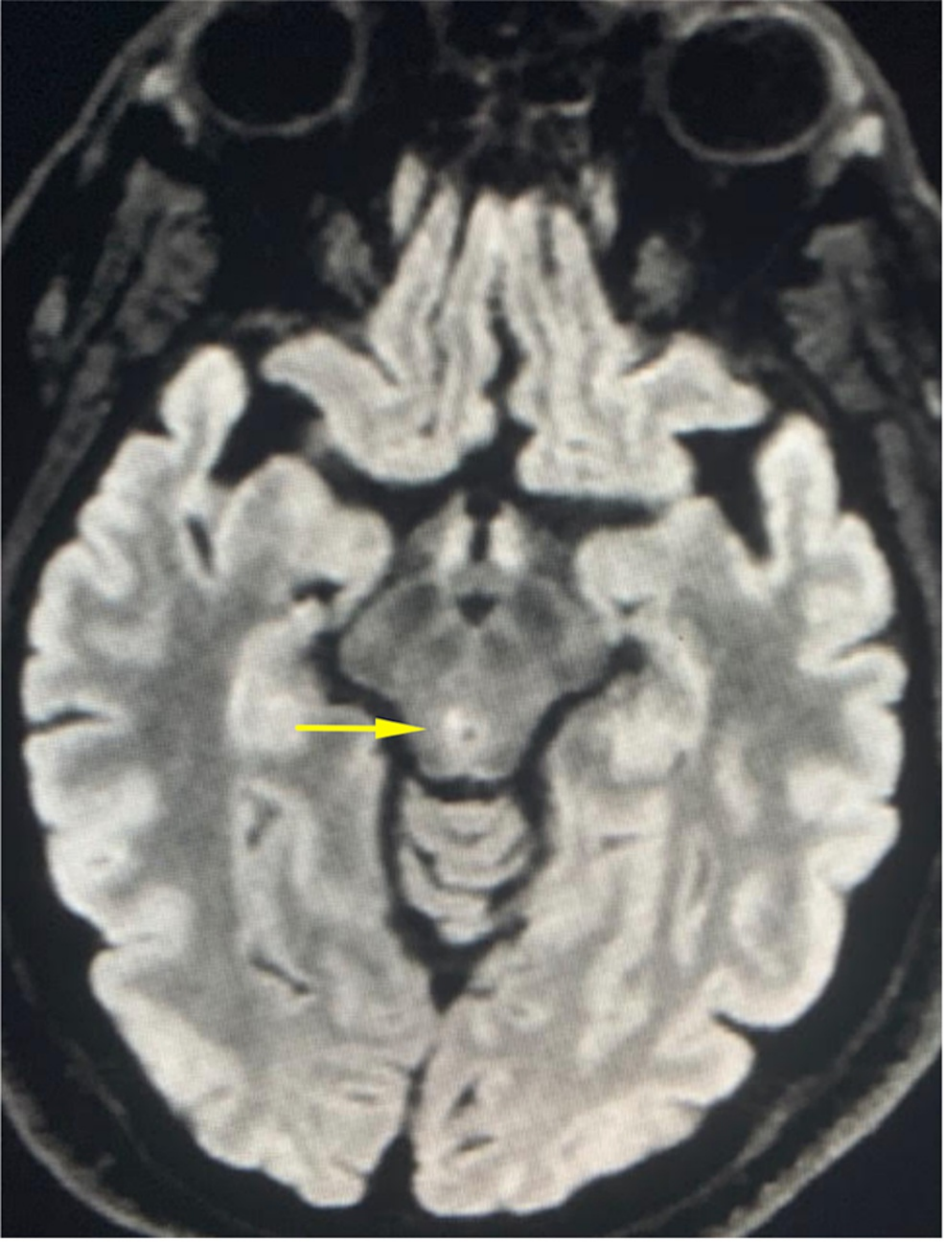
Axial FLAIR demonstrating oculomotor nuclear lesion FLAIR: fluid-attenuated inversion recovery

Serum chemistries upon admission were remarkable for signs of dehydration and cell counts were within normal limits. The patient underwent imaging, which found a 1.5 mm area of restricted diffusion in the region of the oculomotor nucleus suspicious for ischemic stroke (Figure 1). She was treated with a strategy of permissive hypertension throughout the duration of her admission and ocular rehabilitation. Treatment was well-tolerated and without complications. 

Image findings and diagnosis

**Figure 2:**
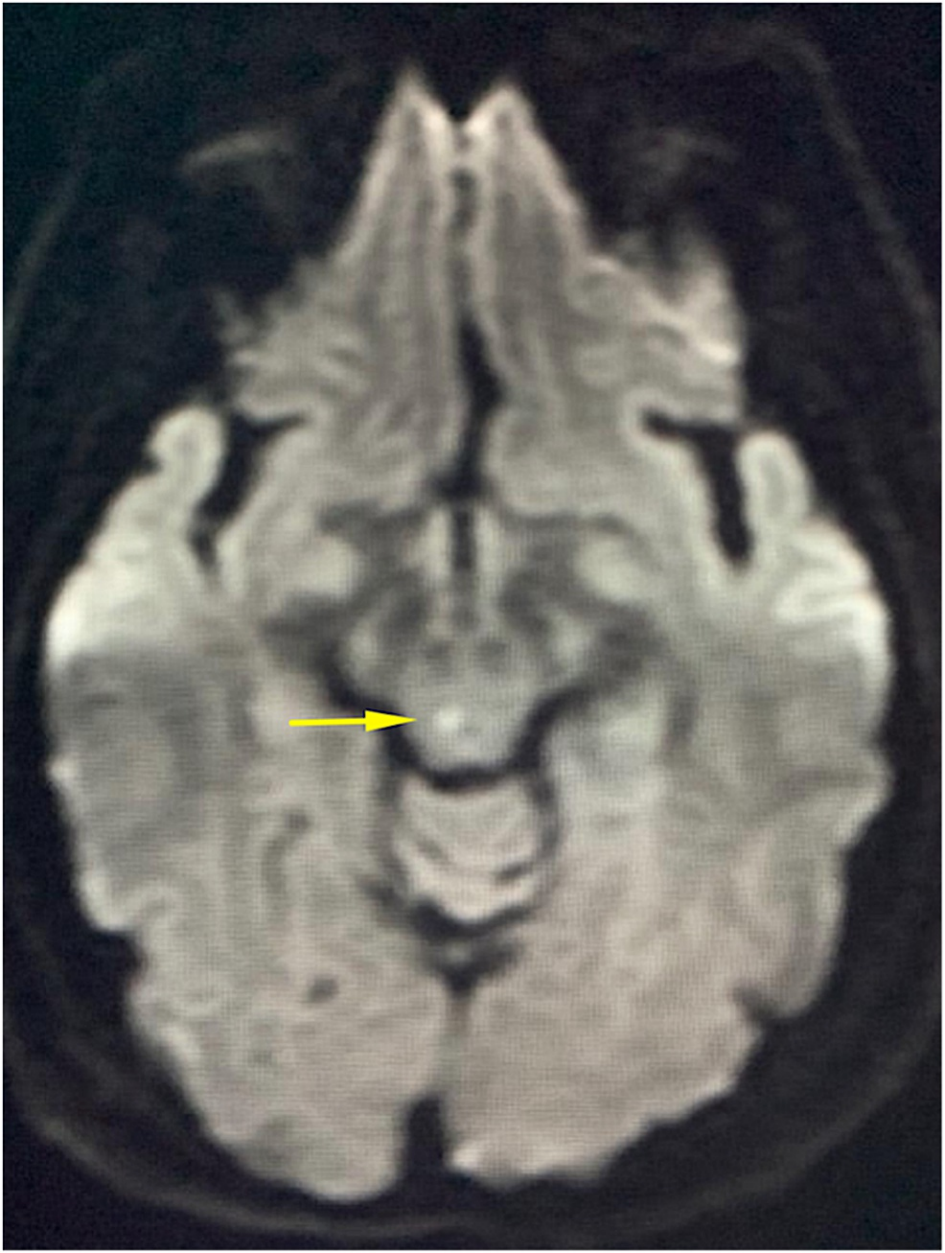
DWI axial view displaying a midbrain intensity DWI: diffusion-weighted imaging

A CT scan of the brain without contrast was performed for evaluation of focal weakness and numbness. Contiguous axial slices from the skull base to the vertex revealed mild involutional and microvascular changes. Small, old lacunar infarcts in the basal ganglia were appreciated bilaterally (Figure 2). The ventricles, cisterns, and sulci were all age-appropriate in size. The orbits were unremarkable and there were no signs of intracranial hemorrhage appreciated. The calvarium was intact and the gray-white matter junction was well distinguishable. No signs of acute infarction were appreciated.

**Figure 3:**
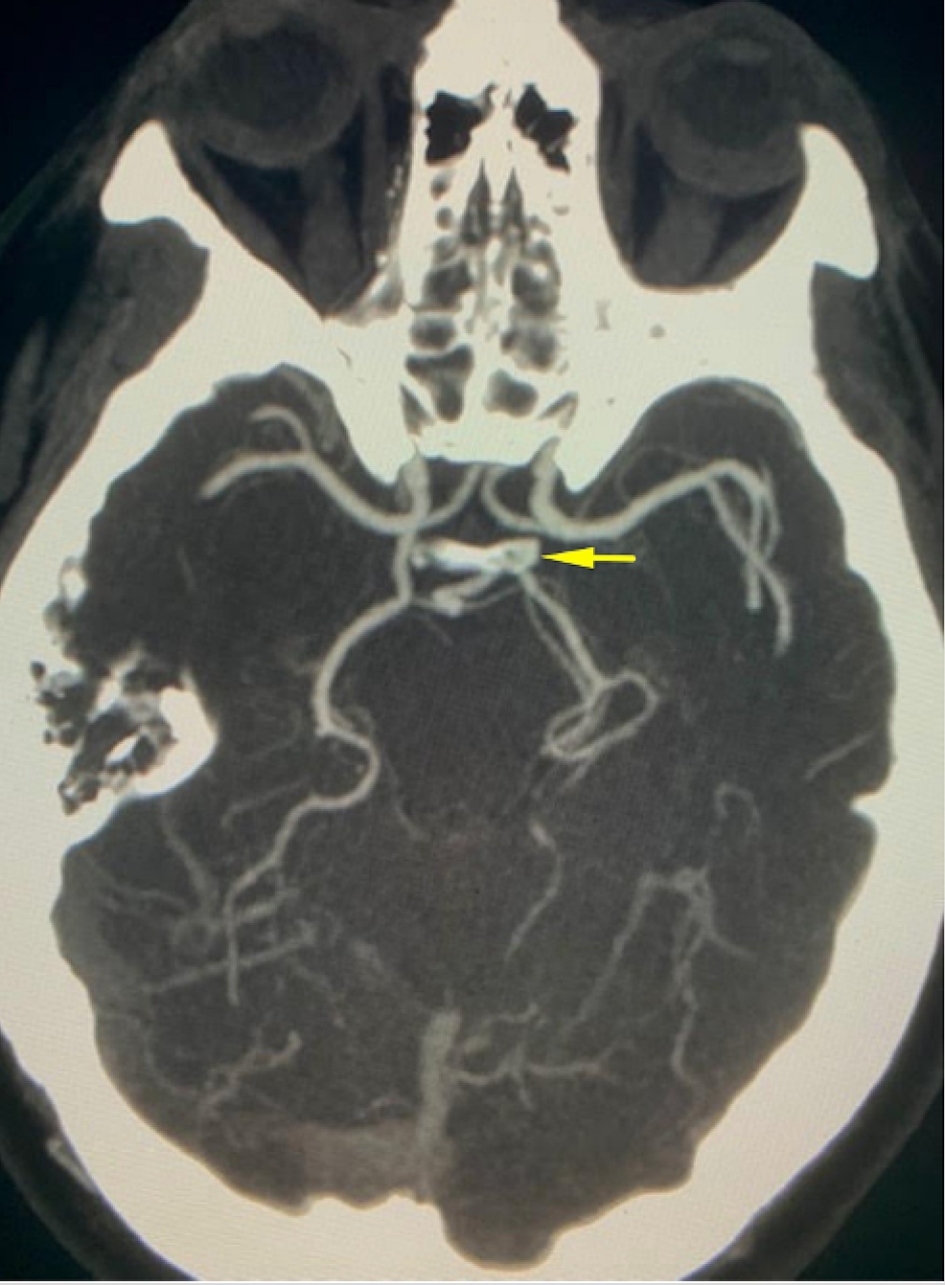
Axial CTA demonstrating fPCA CTA: computed tomography angiography; fPCA: fetal posterior cerebral artery

CT angiography of the neck from the aortic arch to the lower cranial level was performed. There was no evidence of carotid or vertebral stenosis bilaterally. There was, however, fetal origin of the PCA, and enlargement of the pulmonary artery up to 3.3 cm which is consistent with pulmonary hypertension (Figure 3). The rest of the computed tomography angiography (CTA) was unremarkable. 

**Figure 4:**
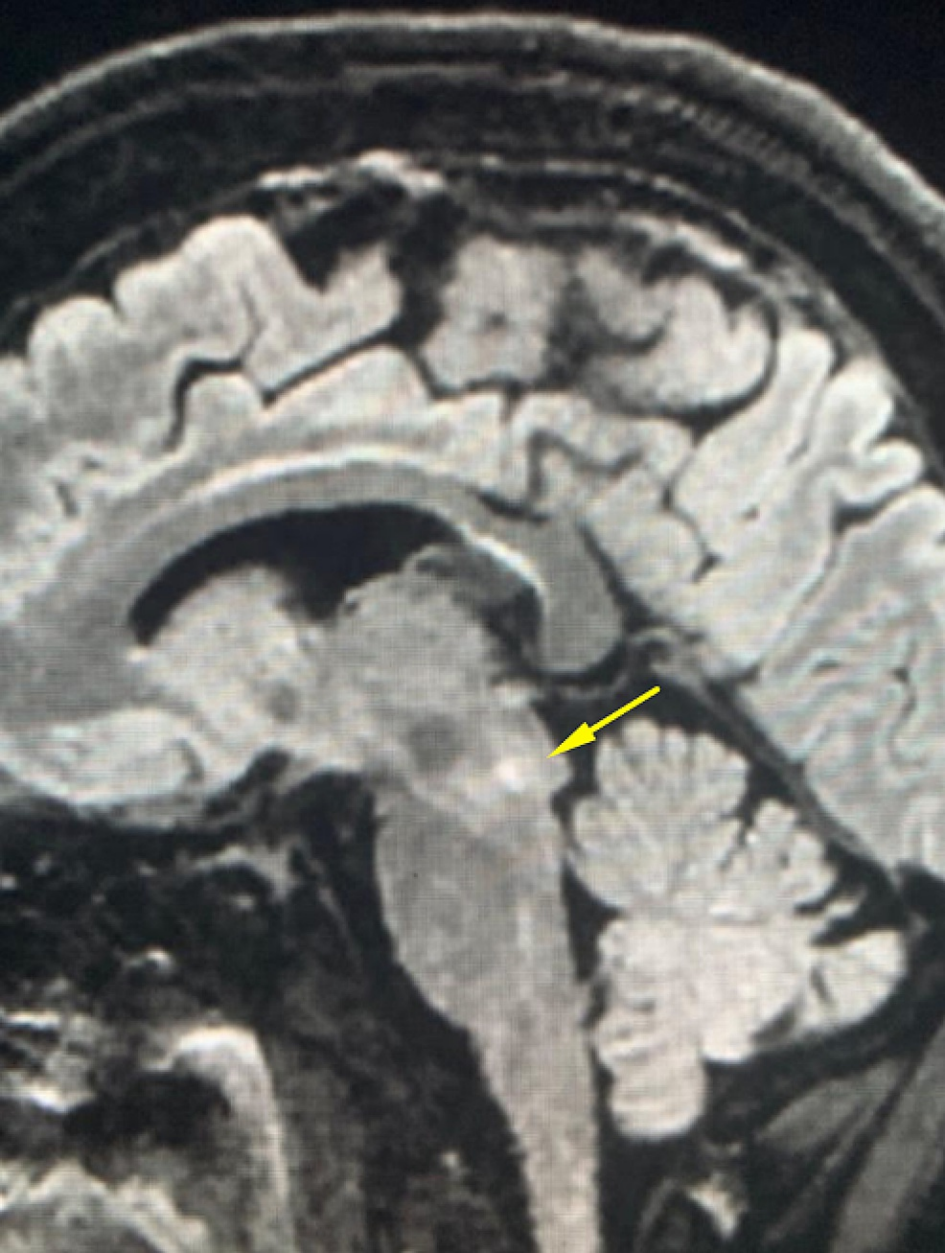
Sagittal FLAIR displaying midbrain lesion FLAIR: fluid-attenuated inversion recovery

Non-contrast magnetic resonance imaging (MRI) was performed with the intention to evaluate for cerebral vascular accidents and source of medial rectus palsy. There were no prior MRI images previously acquired with which radiology could compare. This modality revealed a punctate focus of restricted diffusion at the right midbrain compatible with acute infarction of approximately 1.5 mm (Figure 4). In light of the patient's symptoms, the affected region may involve the oculomotor nerve nucleus. The remainder of the study is unremarkable showing a mild motion artifact at T1 and involutional changes consistent with the patient’s age. 

## Discussion

The circle of Willis (CoW) arises from the connection of the vertebrobasilar/posterior system supplied by the basilar artery, and the ICA/anterior system supplied by both internal carotid arteries. The structure of the brain’s internal vasculature is often taught as being consistent between individuals, but past studies have demonstrated that this is not the case. A complete and symmetrical CoW can be seen in as little as 42.1% of the population [7]. The fPCA seen in our patient is a relatively common variant of the CoW that can arise during fetal development [6]. At the 40 mm developmental stage (eight weeks), the fetal vertebrobasilar system forms bilateral connections with the posterior communicating branches of each PCA. These connections become the future P1 segments of the PCA. The diameter of each vessel contributing to the CoW is the same at this point of fetal development.

In some individuals, the P1 segment remains its fetal size as the rest of the CoW grows. For these individuals, the hypoplastic P1 segment either remains as a low caliber connection between the vertebrobasilar and ICA systems or, in some cases, fails to develop at all, becoming too small to contribute in aid in perfusing the cerebral circulation [8]. Both scenarios are categorized as fPCA anatomical variations [6]. Though there have been many different studies on this particular variant, the medical implications still remain largely unknown and understudied.

In individuals with a complete CoW, posterior cerebral circulation is derived from both the basilar artery and the ICA. Because the fPCA variant compromises the P1 connection between the basilar artery and the PCA, individuals possessing this variant only have one source for ipsilateral posterior cerebral circulation - the ICA [6]. The oculomotor nucleus is located in the medial midbrain, an area that derives its blood supply from the paramedian branches of the basilar artery and the proximal PCA [9]. The P1 segment is the origin of the paramedian arteries [10]. The loss of contribution from the P1 segment in individuals with fPCA may compromise the supply to the paramedian branches. 

Vascular compromises push the brain to display its plasticity in the form of leptomeningeal collateral circulation. Leptomeningeal vessels are low-caliber anastomoses that form between different vascular territories in the brain. Their supply is dependent on the hemodynamic state of the connected territories [8]. Generally, these collateral vessels connect territories supplied by the vertebrobasilar system with the areas supplied by the ICA system. In individuals with fPCA, the tentorium prevents the brain from connecting these two regions by obstructing the formation of leptomeningeal collateral vessels from the cerebellum that would otherwise correct the loss of vertebrobasilar contribution to the PCA’s circulation [8]. Compounded with the loss of the P1 paramedian branches, an argument for increased risk of ischemia in the oculomotor nucleus presents itself.

The neurological symptoms associated with a stroke inform clinicians which areas to focus on when evaluating the neuroimaging. The relative rarity of PCA strokes (20%) makes clinical symptoms important in localizing the lesion [4]. In our patient, the symptoms of diplopia, medial rectus palsy, and pupillary dilatation lead to a focus on the areas supplied by the PCA [4]. The MRI finding of a 1.5 mm area of restricted diffusion in the region of the right oculomotor nucleus supports the overall clinical picture of an ischemic stroke in an area partially supplied by the proximal PCA [9]. Our patient has a previously known unilateral fPCA variant ipsilateral to the MRI finding of a small and specific area of restricted diffusion in the territory of this vascular anomaly [9]. The presence of an fPCA with loss of P1 compounded with the loss of leptomeningeal collateral circulation from the vertebrobasilar system offers a possible explanation for the suspected ischemic changes visualized [8]. The altered blood flow in patients with an fPCA resulting from the ICA becoming the primary vessel supplying the PCA’s distribution adds to this list of hemodynamic conditions that may put the oculomotor nucleus at increased risk for ischemia [8]. The clinical picture established through an extensive evaluation of our patient demonstrates the need for further exploration of the fPCA anatomical variant and its potential implication in suspected ischemic ipsilateral oculomotor nucleus strokes.

## Conclusions

Cerebrovascular accidents are presently the fifth leading cause of death in the United States annually and are the leading cause of long-term disability. Posterior circulation strokes namely including blood flow in the regions of the vertebral, basilar, and PCA represent a large proportion of all strokes. Signs and symptoms demonstrative of strokes involving the PCA include ocular apraxia, ptosis, pupillary dilation, and the classic "down and out" appearance of the ipsilateral eye, along with contralateral muscle weakness, hyperreflexia, and ipsilateral facial numbness. 

With regards to our case, we report fPCA seen in a stroke patient, which can be further classified as either a partial or complete fPCA. This is determined by whether P1, the PCA segment directly arising from the terminal of the basilar artery, is hypoplastic or absent. If absent, the PCA has arisen completely from the ICA. If a hypoplastic segment remains, however, it is known as a partial fetal PCA. In the case of this patient, we report a partial infarction of the oculomotor nucleus with ipsilateral fPCA involvement in a 59-year-old female. Whether or not the presence of fPCA predisposed this individual to ischemic damage, or mediated the outcome in any way, has yet to be determined and would require further investigation. Nonetheless, this case demonstrates an unusual presentation of a cerebral occlusive disease that should be taken into account for suspected posterior circulation strokes.
